# Quality assessment of glucose measurement with regard to epidemiology and clinical management of diabetes mellitus in Germany

**DOI:** 10.3389/fmolb.2024.1371426

**Published:** 2024-03-20

**Authors:** Peter B. Luppa, Michael Zeller, Marija Pieper, Patricia Kaiser, Nathalie Weiss, Laura Vierbaum, Guido Freckmann

**Affiliations:** ^1^ Institut für Klinische Chemie und Pathobiochemie, Klinikum rechts der Isar der Technische Universität München, Munich, Germany; ^2^ INSTAND e.V., Gesellschaft zur Förderung der Qualitätssicherung in Medizinischen Laboratorien e.V., Düsseldorf, Germany; ^3^ Institut für Diabetes-Technologie, Forschungs- und Entwicklungsgesellschaft mbH an der Universität Ulm, Ulm, Germany

**Keywords:** EQA schemes, diabetes prevalence, diabetes management, ISO 15197, glucose in plasma, HbA1c in whole blood

## Abstract

**Background:**

During the last decade, Germany has seen an increased prevalence and a redistribution from undetected to diagnosed diabetes mellitus. Due to this substantial epidemiological development, the number of people with documented type 2 diabetes was 8.7 million in 2022. An estimated two million undiagnosed subjects are to be added. Beyond that, the life expectancy of diabetic subjects is increasing due to more responsive health systems in terms of care. Possible reasons include improved screening of at-risk individuals, the introduction of HbA1c for diagnosis in 2010, and the higher use of risk scores. Additionally, quality aspects of the laboratory methodology should be taken into consideration.

**Methods:**

Epidemiology and clinical management of diabetes in Germany are presented in the light of publications retrieved by a selective search of the PubMed database. Additionally, the data from German external quality assessment (EQA) surveys for the measurands glucose in plasma and HbA1c in whole blood, reviewed from 2010 until 2022, were evaluated. Above this, data concerning the analytical performance of near-patient glucometer devices, according to the ISO norm 15197:2013, were analyzed.

**Results:**

Two laboratory aspects are in good accordance with the observation of an increase in the diabetes mellitus prevalence when retrospectively reviewing the period 2010 to 2022: First, the analytical performance according to the ISO norm 15197:2013 of the glucometer devices widely used by patients with diabetes for the glucose self-testing, has improved during this period. Secondly, concerning the EQA program of INSTAND, the number of participating laboratories raised significantly in Germany. The spreads of variations of the specified results for plasma glucose remained unchanged between 2010 and 2022, whereas for HbA1c a significant decrease of the result scattering could be observed.

**Conclusion:**

These retrospectively established findings testify to an excellent analytical quality of laboratory diagnostics for glucose and HbA1c throughout Germany which may be involved in a better diagnosis and therapy of previously undetected diabetes mellitus.

## Introduction

Diabetes mellitus is a group of common endocrine diseases characterized by sustained high plasma glucose and elevated whole blood glycated hemoglobin (HbA1c) concentrations and resulting clinical signs of persistent hyperglycemia. The chronic and untreated life-threatening disease is due to either pancreatic lesions resulting in impaired insulin secretion or peripheral cells becoming unresponsive to insulin to a variable degree and its subsequent metabolic effects (so-called peripheral insulin resistance) (Brutsaert, 2023). The vast majority of affected patients suffer from type 1 and type 2 diabetes. The high worldwide burden of diabetes has adverse health effects on affected individuals, but also economic impacts on the global healthcare systems.

In Germany, seven million people had documented type-2 diabetes in 2015. In the same year, 32,000 children and adolescents, as well as 340,000 adults, had type 1 diabetes. Due to the increasing prevalence data, the number of people with documented type 2 diabetes was expected to reach 8.7 million in 2022 (Tönnies et al., 2019).

Worldwide, diabetes mellitus and the healthcare resources required to treat the disease result in challenging high socio-economic costs. With approx. forty billion €, Germany has the fourth highest healthcare expenditure on diabetes. Healthcare costs for affected patients are around twice as high as for comparable people without diabetes. A large proportion of healthcare expenditure is spent on treating secondary diseases of diabetes. Sophisticated disease management programs can limit the increase in this expenditure (Deutsche Diabetes Gesellschaft, 2023).

Laboratory medical examinations are of great importance in the diagnosis and subsequent disease management of diabetes mellitus (Schleicher et al., 2022). Blood or plasma glucose measurement has long been a proven analytical method performed in the central laboratory, but also near-patient blood glucose measuring devices, which, if subjected to close-controlled quality assurance measures, allow highly accurate determinations of plasma glucose. Furthermore, in the last decade, HbA1c has emerged as a long-term diagnostic parameter in addition to the already-known assessment for glycemic control in people with diabetes. It may complement the determination of glucose in a diagnostically helpful way. The essential role of HbA1c is that it can be used to make a statement about the blood glucose control of the last 8–12 weeks and can thus be applied as a therapy control to reduce possible consequential damage (Weykamp, 2013). In addition to diagnosing new-onset diabetes mellitus, the lifelong monitoring of glucose metabolism is another vital pillar of treating this disease. Most patients carry out this measurement by themselves using glucometers with unit-use test strips daily. This so-called self-measurement of blood glucose (SMBG) has recently been supplemented by continuous glucose monitoring (CGM) systems (Lin et al., 2021), which involves continuous measurement of glucose in the interstitial body fluid. In addition, CGM-controlled and partially automated insulin dosing systems are already on the rise.

Ongoing improvements in SMBG/CGM analytics, insulin injection technology, and data management have evolved into a novel modern form of diabetes treatment alongside education/counseling and adequate drug therapy. Physicians in diabetology-focus practices, outpatient clinics, and hospitals are thus provided with more and more data to assess and optimize the quality of the individual patient’s diabetes situation (Kravarusic and Aleppo, 2020).

The accuracy and precision of laboratory parameters undoubtedly have a direct impact on diagnosis and patient care. All measurements, including those of the pivotal parameter plasma glucose concentration, are subject to an inherent measurement uncertainty (Petersmann et al., 2022). Analytical efforts should, therefore, always aim to reduce the measurement uncertainty in order to meet the requirements for the diagnosis and treatment of diabetes. Such efforts have been observed in recent years, including for HbA1c. For this parameter, the permissible relative root mean square measurement error in EQA schemes was reduced from ±10% to ±3% (Bundesärztekammer, 2023). This should improve the analytical differentiation between the important HbA1c cutoff values of 39 and 48 mmol/mol (5.7% and 6.5%).

The aim of this article is, therefore, to highlight the changes in the epidemiology and clinical management of diabetes mellitus that have been achieved over the last 2 decades and to causally relate them to the advances in analytical capabilities and improved quality assurance measures, here, in particular, the External Quality Assessment (EQA) schemes for the measurands glucose and HbA1c, executed in Germany.

The International Federation of Clinical Chemistry and Laboratory Medicine (IFCC) defines EQA as laboratory performance and method evaluation for regulatory purposes, focusing on participating laboratories’ or physicians’ education and support (Blasutig et al., 2023). EQA primarily evaluates the analytical performance of participants concerning measurands by comparison to a target consensus value (CV) within a method split or by comparison of all methods to a reference method value (RMV). In Germany, EQA schemes are classified as regulatory based on the valid at the time “Guideline of the German Medical Association for the Quality Assurance of Laboratory Medical Examinations (Rili-BÄK)” (Bundesärztekammer, 2023). The Society for Promoting Quality Assurance in Medical Laboratories (INSTAND), Düsseldorf, and the Reference Institute for Bioanalytics (RfB), Bonn, are accredited organizations performing EQA schemes in laboratory medicine.

Strict quality standards for IVD manufacturers are also mandatory for a premarket evaluation to ensure the measurement quality of the respective device. The international standard ISO 15197 (see below) is a norm applied to blood glucose monitoring systems.

## Materials and methods

### Epidemiological data for diabetes mellitus, gestational diabetes

Epidemiology and clinical management of diabetes in Germany are presented in the light of publications retrieved by a selective search of the PubMed database (search terms were *diabetes mellitus type 1 AND diabetes mellitus type 2 AND epidemiology AND mortality AND clinical management)*, as well as by the annual healthcare reports of the German Diabetes Society (Deutsche Diabetes Gesellschaft, DDG) and its pertinent guidelines (including gestational diabetes).

### EQA data for glucose and HbA1c

The second data source was the EQA surveys for the measurands glucose and HbA1c, conducted by INSTAND, from 2010 to 2022. Each survey (synonym “ring trial”) is offered six times per year. For the statistical analysis of the quantitative results for glucose and HbA1c, only the last EQA scheme of the year, conducted in October, was used since it was regularly the largest one regarding the number of participants. Concerning our analysis, the EQA schemes discussed are evaluated as follows: EQA #100 (clinical chemistry parameters, including glucose): RMV; #145 (HbA1c): RMV; #800 (glucose POCT): CV. In each EQA survey, two samples with different concentration levels (randomly assigned as samples A and B) are delivered to the participants. These concentrations are chosen to be within the linear measurement range of all possible methods used by the participants.

The total numbers per year were analyzed to determine the dynamics of the participation. We also used the respective RfB information for the corresponding ring trials. In general, all participants must report quantitative results together with additional information on the test kit provider and laboratory equipment used via the online portal of the respective reference institution. Table 1 summarizes the number of participants and applied methods for the INSTAND EQA schemes.

**TABLE 1:** Characteristics of INSTAND’s EQA schemes. RMV indicates the use of a respective reference method value for evaluation, whereas CV stands for consensus value mode.

EQA scheme measurand, additional description	Code #	Average number of participants per survey^a^	EQA evaluation mode	Number of evaluated device types/methods
Glucose as part of the clinical chemistry EQA	100	657	RMV	8/2
Glucose POCT	800	706	CV	28/2
HbA1c	145	701	RMV	17/3 Proportions of methods: 61% immunological; 25% HPLC; 5% affinity chromatography

^a^ Per year, there are six EQA surveys for each scheme.

INSTAND offers EQA schemes for glucose in plasma as part of the clinical chemistry panel (#100) or separate as POCT glucose samples (#800). We analyzed the value scattering of the respective results of the EQA #100 (glucose oxidase (GOD) and hexokinase/Glc-6P-DH method) and #800 (GOD and Glc-DH method) throughout the whole period 2010–2022 without differentiation of the methods applied. For HbA1c as the second diabetes measurement, the EQA organization has used fresh whole blood samples since 2015, with target values assigned with the IFCC reference measurement procedure. Therefore, we analyzed this ring trial only in 2015–2022. Here, we differentiated between affinity chromatography, ion-exchange HPLC, and immunological methods.

### Statistical methods applied

**FIGURE 1:**
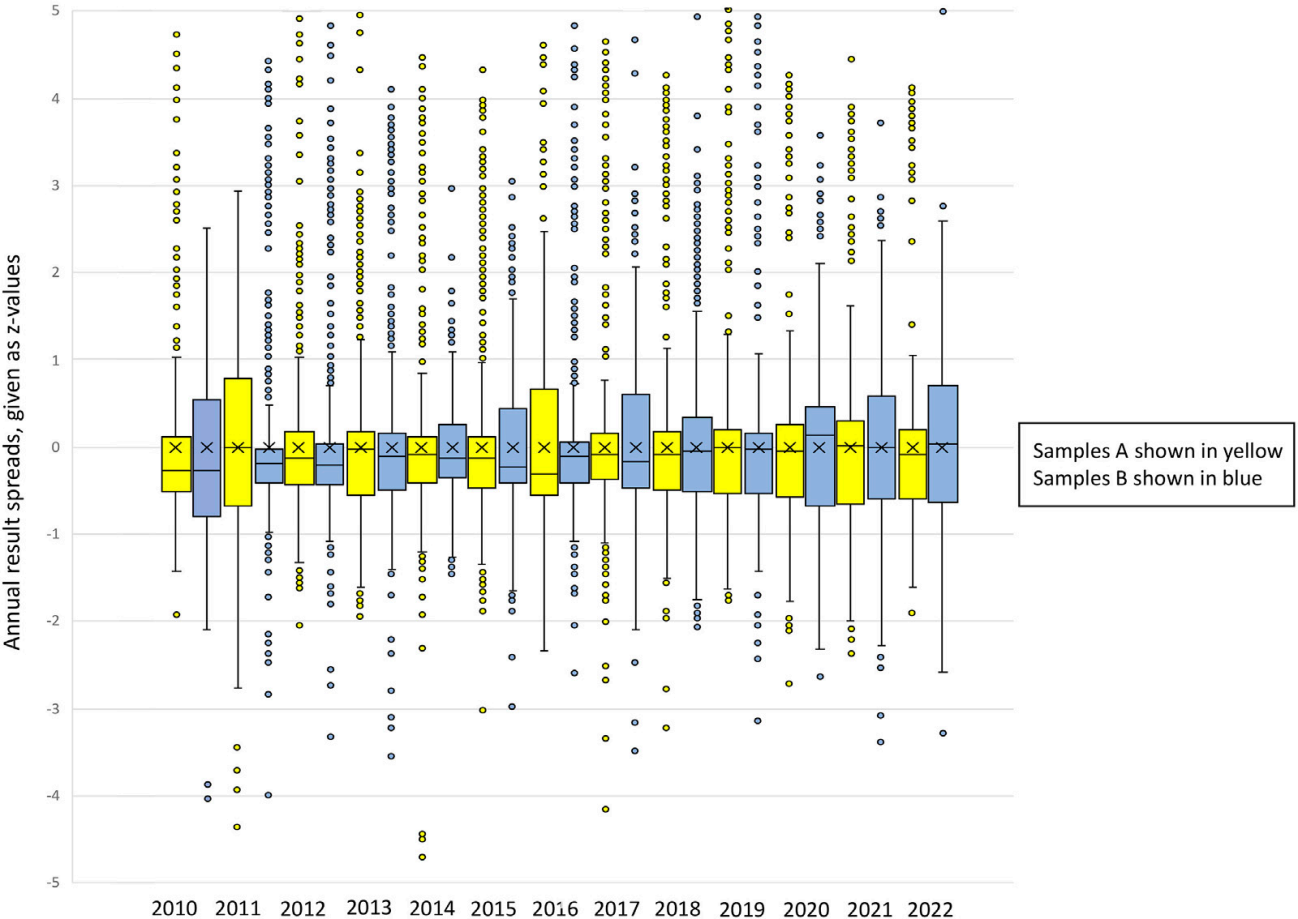
Box-and-whisker plots of the annual result spreads for EQA scheme #800 (POCT glucose), given as z-values. Samples A and B are shown for each year. The total sum of results evaluated for this EQA scheme was 17,125. Description of the box-and-whisker plot: The middle line represents the median, whereas the x in the box represents the mean of the z-values. The box includes the lower and the upper quartiles (25%–75%). The whiskers show the minimum and maximum values (±1.5 x interquartile range (IQR)). Single points represent outliers.

The result data of the participants for the respective EQA scheme measurand were recalculated by z-scoring. The z-values are the numerical values of the positive or negative standard deviations from the respective CV (for #800) or RMV (for #100 and #145). The resulting z-value ranges of the EQA participants give an impression of the scatter of the individual measured values and are depicted as box-and-whisker plots. The middle line represents the median, whereas the x in the box represents the mean of the z-values. The box includes the lower and the upper quartiles (25%–75%). The whiskers show the minimum and maximum values (±1.5 × the interquartile range (IQR)). The extremes (below or above ±1.5 × IQR) were excluded, as these reported values were mainly compromised by gross errors (sample mix-up, wrong unit, etc.). However, in Figure 1, the outliers are also shown to illustrate the wide scatter of the individual z-values.

Possibly significant changes in the value range over time and in the number of participants of the respective EQA schemes were then investigated by linear regression analysis (least squares method). The degree of association is represented by the coefficient of determination *R* ^2^, measured on a scale ranging from −1 through 0 to +1. Complete correlation between two variables being expressed by either +1 or −1. The significance level for the trend line slope >0 was set to *p* < 0.05. All statistical data were calculated using the Microsoft Excel add-in Abacus 3.0, LABanalytics GmbH, Jena, Germany.

### Determination of the analytical performance of glucometers according to the ISO standard 15197:2013

A comparative survey and meta-analysis of publications from 2012 to 2022 was performed. A Medline search in this time frame selected these publications. Search terms were *glucometer OR blood glucose monitoring system OR BGMS OR plasma glucose analysis AND ISO 15197*. In brief, ISO 15197:2013 claims the following minimum requirements: First, at least 95% of blood glucose monitoring system (BGMS) results from three different strip lots have to be within ±15 mg/dL at glucose concentrations <100 mg/dL or within ±15% at ≥100 mg/dL, being compared to a traceable laboratory method. Second, in a consensus (Clarke) error grid analysis, at least 99% of results must be within zones A and B. The different authors checked these requirements.

## Results

### Prevalence and redistribution of undiagnosed people with diabetes in Germany

#### General prevalence data

Using the search terms, 122 hits in Medline were found. Most informative for understanding the epidemiological situation in Germany were the annual health reports of the DDG since 2010. For years, these health reports (analysis period 2010–2022) have noted an increasing prevalence of diabetes in the German population. As a result, the number of people with type 2 diabetes in Germany in 2022 rose to approximately 8.7 million (Tönnies et al., 2019); the number of unreported cases could be estimated at two million (Deutsche Diabetes Gesellschaft, 2023). By comparison, the number of diagnosed diabetes cases in 2015 was seven million.

This increase in diabetes prevalence in Germany was predominantly accounted for by subjects aged 65 years and older and those with low educational status, a high body mass index (>30 kg/m^2^), and a low physical activity profile (Heidemann et al., 2016). The authors additionally pointed out that the life expectancy of persons with diabetes might have increased more in the last 2 decades than the general population due to more responsive health systems in diagnostics and care (Tönnies et al., 2021). Other potential causes for the increase in prevalence include earlier identification of affected patients using the laboratory parameter HbA1c in whole blood for diagnosis and the increased clinical use of diabetes risk scores (Heidemann et al., 2016). Together with the observation that there has been a decrease in undetected diabetes since 2012, these results suggest that there has been a redistribution from undetected diabetes to diagnosed diabetes in recent years (Deutsche Diabetes Gesellschaft, 2020).

#### Screening of risk factors and risk scores for type 2 diabetes

To identify patients at increased risk for type 2 diabetes, screening of asymptomatic individuals based on risk factors or risk scores has been recommended in the practice recommendations of the DDG for years (Heidemann and Scheidt-Nave, 2017): Detecting prediabetes based on fasting plasma glucose, 2-h oral glucose tolerance test (OGTT) plasma glucose, or HbA1c. Although the benefits of earlier diagnosis of diabetes are still somewhat unclear, the benefits of lifestyle intervention in individuals with prediabetes have led the US Preventive Services Task Force to recommend screening for prediabetes and type 2 diabetes in nonpregnant adults 35–70 years of age who are overweight or obese (Goffrier et al., 2017; US Preventive Services Task Force, 2021). The task force concludes with moderate certainty that screening for prediabetes and type 2 diabetes and offering or referring patients with prediabetes to effective preventive interventions has a moderate net benefit.

An indirect approach to determining the risk of diabetes is using risk scores. These allow the estimation of the statistical probability that a person will develop type 2 diabetes in a defined period. Prognostically relevant risk scores allow quantification of risk using a combination of multiple risk parameters and can assist in accurately determining the disease risk for individuals (Deutsche Diabetes Gesellschaft, 2023; Lind et al., 2013).

#### Gestational diabetes

Since 2012, the German maternity guidelines have recommended a systematic screening program for gestational diabetes using an OGTT (Schäfer-Graf et al., 2018). One of the prerequisites for an effective laboratory screening program is an accurate and precise determination of the glucose concentration in venous plasma. Whenever it is impossible to rapidly test the glucose concentration from whole blood, stabilized blood has to be sent to the analyzing laboratory. The progress made within the last decade was the finding that an effective pre-analytical glycolysis inhibition can only be achieved by using sodium fluoride combined with an acidic citrate buffer (Gambino et al., 2009). Without citrate buffering in the blood collection tubes, false low glucose concentrations may occur, which leads to undetected gestational diabetes.

### Results from EQA schemes concerning the total number of participants and the spread of result variations for glucose and HbA1c

#### Number of participants

In Germany, INSTAND and RfB offer EQA schemes for glucose in plasma as part of the clinical chemistry panel or separate as POCT glucose samples. For the second diabetes measurand, HbA1c, both organizations use fresh whole blood samples with target values assigned with the IFCC reference measurement procedure since 2015.

In 2010–2022, the POCT glucose EQA schemes significantly increased participation in both EQA organizations. In contrast, in the HbA1c dedicated EQA schemes, a substantial increase in the number of participants could be observed only for RfB. For INSTAND, the number of participants decreased between 2015 and 2016. This was due to the shift of the sample matrix from processed to fresh whole blood and the resulting changes in the delivery of samples. Results are summarized in Table 2 and can be retrieved from the Supplementary Figures S1A–D.

**TABLE 2:** Linear regression analysis concerning the number of participants in the EQA schemes conducted by INSTAND and RfB for POCT glucose and HbA1c.

Measurand	EQA organization	Number of investigated years (2010–2022)	*R* ^2^	*p*
POCT glucose	RfB	13	0.932	<0.01
POCT glucose	INSTAND	13	0.664	<0.01
HbA1c	RfB	13	0.614	<0.01
HbA1c	INSTAND	13	0.527^a^	−

^a^ R = −0.726. Not evaluable due to a change of the offered EQA, material.

#### Spread of variations of the EQA results

For a better understanding, Figure 1 portrays the spread of result variations throughout the years for the EQA scheme #800 glucose POCT. The outliers are also shown here to illustrate the wide range of the individual z-values. The total sum of results evaluated for this EQA scheme was 17,125.

We found that no significant narrowing of the value spreads could be observed for the glucose EQA schemes #100 and #800. The correlations found showed no significant positive or negative slope. Interestingly, the spreading width of the glucose results in #100 was constantly lower than the width of results in #800. This testifies to a constant high quality of the laboratory analysis in Germany.

The situation is different for the measurand HbA1c. Here, the samples given out by INSTAND have been commutable since 2015, when a new whole blood sample matrix was introduced. In the shorter observation period 2015–2022, significant decreases of result scattering could be observed for two of the three different methods for the analysis of HbA1c: ion-exchange HPLC and immunological methods. Only the affinity chromatography method showed no significant negative slope. The respective statistical data can be found in Table 3.

**TABLE 3:** Linear regression analysis concerning the calculated z-values, summarized for samples A and B, representing the variability of the individual results in the INSTAND EQA schemes for glucose (#100 and #800) and HbA1c (#145).

Measurand	Number of investigated years (2010/2015–2022)	*R* ^2^	*p*
Glucose clinical chemistry (#100)	13	0.007	0.689
Glucose POCT (#800)	13	0.066	0.205
HbA1c (#145) affinity chromatography	8	0.120	0.188
HbA1c (#145) ion-exchange HPLC	8	0.245	0.051
HbA1c (#145) immunoassay methods	7^a^	0.369	0.021

^a^ The year 2015 was excluded as the number of participants was exceptionally low due to the change in the EQA, material supplied.

A complete set of the result spreads for the EQA schemes, shown as box-and-whisker plots, can be retrieved from Supplementary Figures S2–S6.

### Data from international studies concerning the analytical performance of glucometers

Sufficiently robust BGMS are a prerequisite for appropriate and safe blood glucose self-monitoring in patients with diabetes. The measurement accuracy of glucometer devices significantly impacts the quality of clinical care and therapy adjustment for these patients (Jendrike et al., 2019). It can be regarded as proven that more significant errors in SMBG devices lead to greater predicted risks of undetected hypoglycemia (Breton and Kovatchev, 2010). Strict accuracy criteria are therefore mandatory for a premarket evaluation to ensure the measurement quality of BGMS systems. These criteria are defined in the international standard ISO 15197 (“*In vitro* diagnostic test systems—Requirements for blood-glucose monitoring systems for self-testing in managing diabetes mellitus”). This standard, first published in 2003 (International Organization for Standardization, 2003), calls for several quality requirements, among them analytical performance evaluations, to guarantee safe and reliable glucose measurements.

Regarding analytical performance, requirements on the accuracy of the respective system (device plus glucose strips) are described in detail, including evaluation design and minimum accuracy criteria. In 2013, a revised version of the norm was published with significant changes like additional stringent accuracy criteria and changes in the testing procedure (International Organization for Standardization, 2013). These criteria were already described in Materials and Methods.

**FIGURE 2:**
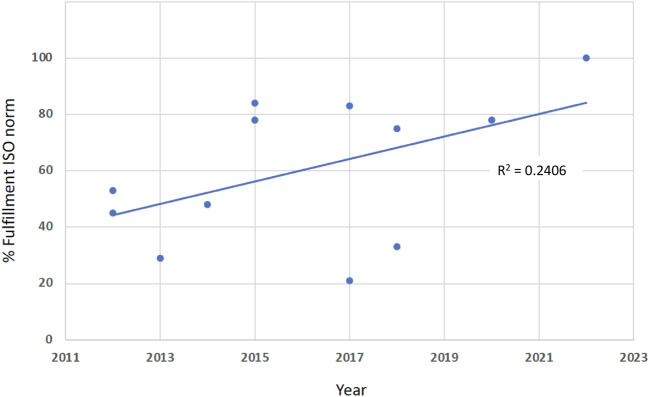
The percentages of fulfillment of the EN ISO norm 15197 (*In vitro* diagnostic test systems - Requirements for blood-glucose monitoring systems for self-testing in managing diabetes mellitus), found in various published studies, are increasing between 2012 and 2022 (*R* ^2^ = 0.2406).

Between 2010 and 2020, a series of methodological studies deals with the compliance of various glucometer systems with the ISO 15197 criteria. Using the search terms, we found 191 hits in Medline; 12 studies were adequate to answer our question. Table 4 displays the percentages of tested reagent system lots that fulfilled the current ISO norm 15197:2013 system accuracy criteria. It can be stated that the percentages increased continuously. This can also be seen in Figure 2. The regression line has an *R* ^2^ of 0.2406. Additionally, in an extensive literature review, 58 studies with 143 different SMBG systems between the years 2010 and 2017 were evaluated for accuracy. It was shown that newer meters were more likely to pass the ISO 15197:2013 standards (King et al., 2018).

**TABLE 4:** Publications showing percentages of tested reagent system lots that fulfill system accuracy criteria of ISO 15197:2013.

Study author, publication year	Number of tested devices/lots	% Fulfillment of ISO 15197:2013
Baumstark et al. (2012)	20	45
Freckmann et al. (2012)	34	53
Brazg et al. (2013)	21	29
Hasslacher et al. (2014)	27	48
Link et al. (2015)	12	84
Freckmann et al. (2015)	27	78
Yu-Fei et al. (2017)	19	21
Baumstark et al. (2017)	18	83
Jendrike et al. (2018)	12	75
Klonoff et al. (2018)	18	33
Pleus et al. (2020)	18	78
Pleus et al. (2022)	4	100

## Discussion

### German EQA results concerning analytical quality for glucose and HbA1c and analytical performance of glucometers

The survey for both glucose EQA schemes showed no significant change in the spread of result variations over 13 years. For the HbA1c survey, however, there was a significant tendency towards narrowed result spreads, which could be seen in the two methods with the highest number of participants (ion-exchange HPLC and immunological methods), where the affinity chromatography method showed no significant change over time.

During the observation, the POCT glucose EQA schemes showed significant increases in participating laboratories and diabetes-specialized ambulances in both EQA organizations. In contrast, in the HbA1c dedicated EQA schemes, a significant increase in the number of participants could be observed only for RfB. This can be seen as a sign of a consistently good analytical quality of laboratory diagnostics throughout Germany, which helps clinicians improve diagnostic and follow-up strategies for patients with diabetes.

The findings for the glucose EQA surveys #100 and #800, however, must be seen against the background that the samples delivered by the EQA organization still suffer from a specimen stability challenge (Wang et al., 2020). The reason is the instability of fresh blood samples, leading to the favored delivery of stabilized sample matrices. In particular, for #800, the matrix effects of such stabilized samples could result in substantial differences in results between the different POCT systems. Therefore, the EQA evaluation can only be carried out according to the consensus value (CV) and not according to the reference method value (RMV) mode.

As depicted in Table 4, the percentages of tested reagent systems that fulfilled the system accuracy criteria of the EN ISO norm 15,197:2013 increased continuously within the last 2 decades. This testifies to a better analytical quality of glucose measurements within the framework of BGMS and may also be linked to a better diagnosis of previously unknown affected patients. However, it must be mentioned here that the published devices do not necessarily reflect the entire IVD market.

Nevertheless, hypothetical patient scenarios (Eichenlaub et al., 2023) can convey to healthcare professionals and patients a novel understanding of the clinical impact of BGMS accuracy. Despite the standardization of accuracy assessment procedures and requirements, the reliability of the BGMS can still be improved to prevent any adverse clinical events. These are, for example, delayed therapy adjustment, hyperglycemia due to excessive food intake, ketoacidosis, and hypoglycemia due to overcorrection.

Another important point that should be mentioned when evaluating the analytical performance of BGMS is that the reference measurement procedures used for comparison in studies have a considerable impact on the resulting measurement accuracy of BGMS. Since there are systematic differences between the manufacturers’ reference measurement procedures used for BGMS calibration and accuracy assessment, this may have potential implications for therapy for patients with diabetes. Therefore, further harmonization of test procedures is desired by various authors to continue the encouraging trend of ever-improving diagnostic capabilities (Freckmann et al., 2022).

What we expected for our EQA HbA1c results, corresponds to the international EurA1c study from 2018: Concerning the analytical performance of HbA1c measurements, this study examined the analytical quality for HbA1c in 2,166 laboratories in 17 different European countries (EurA1c Trial Group, 2018). The results were evaluated according to the criteria of the IFCC model for analytical quality targets. There were two groups in the study. One group received fresh whole-blood samples, and the other lyophilized hemolysate samples. Only one of 20 participating laboratories did not meet the IFCC criterion. Substantial differences between countries and between manufacturer groups were seen by the study group. Germany was in the group with fresh whole-blood samples and achieved a very good result with an IFCC bias of −0.2. Overall, there were no major differences between the fresh whole-blood group and the group using lyophilized hemolysate samples. The findings are in accordance with our results, showing consistently good accuracy of the different HbA1c methods over the entire observation period.

### Systematic screening for gestational diabetes—situation since 2012

The German maternity guidelines recommend systematic screening for gestational diabetes using an oral glucose tolerance test since 2012 (Schäfer-Graf et al., 2018). As a result, the prevalence of gestational diabetes significantly increased from 4.6% to 6.8% (2018: 51,318 cases) from 2013 to 2018 (Reitzle et al., 2021). The number continues to grow until 2021 with a prevalence of 8.5%, equivalent to several 63,000 cases. This increase can be explained by several factors: First, in the rise in the age of pregnant women; secondly, by an increase of the pre-conceptional body mass index of the fertile group of women; and finally, by the screening effort itself as a health insurance benefit (Deutsche Diabetes Gesellschaft, 2023). However, laboratory diagnostics also made its contribution. Therefore, analytical aspects are worth mentioning here: To avoid false negative glucose results due to a pronounced metabolic breakdown of the measurand in whole blood (Gambino et al., 2009), a national guideline recommends analyzing the glucose concentration immediately from freshly drawn venous blood by use of quality-assured POCT devices or to use citrate-buffered NaF-tubes when the samples have to be shipped to a laboratory site (Neumaier et al., 2015). This has most likely a positive impact on the false negative results.

### Possible link between reduced diabetes mortality, better glycemic control, and an increase in diabetes prevalence by improved laboratory analytics?

Diabetes is a common cause of increased mortality. A recent study on more than 50,000 Spanish individuals impressively showed again that diabetes is associated with a higher premature mortality rate from cardiovascular disease, cancer, and noncardiovascular non-cancer causes compared with the general population (Baena-Díez et al., 2016). Against this background, Chen et al. (Chen et al., 2020) investigated the link between the mortality rate and the prevalence of diabetes mellitus in Caucasian populations. The authors found a significant decline in all-cause mortality since 2000. They concluded that this falling mortality would likely lead to an increasing prevalence despite a stable or even declining incidence of diabetes. They discuss the same public health-related factors as mentioned above, which reduce mortality risk factors. Among them is the optimization of the quality of the analytical techniques with improvements in glycaemic control: Better analytics leads additionally to a higher redistribution rate of undiagnosed diabetes.

Heidemann et al. (Heidemann et al., 2016) further explained factors for the rising prevalence of diabetes in Germany. The authors listed several factors that may be jointly responsible for this observed shift: Increased life expectancy in people with diabetes compared to the general population and the broad clinical application of risk score protocols. Additionally, the drawdown of the cut-off value for the fasting glucose concentration, as proposed by the American Diabetes Association (ADA) in 1997 (Expert Committee on the Diagnosis, 1997) and followed by the WHO 2 years later, combined with the introduction of HbA1c as a valid diagnostic parameter potentially contributed to the observed earlier diabetes diagnosis. However, it must be stated that the diagnostic application of this measurand requires optimized laboratory analytics in terms of accuracy.

Another possible link between better glycemic control of patients with diabetes and optimized laboratory analysis of HbA1c by use of EQA schemes can be deduced from a study by Tollånes *(* Tollånes et al., 2020). The combination of validated patient data and EQA data showed that patients in offices of general practitioners who participate in HbA1c EQA surveys have lower HbA1c levels. The authors conclude that accurate HbA1c results may improve the diabetes care of the affected patients.

A further Norwegian study investigated various factors that can lead to over- and undertreatment of hyperglycemia. The study examined 10,233 individuals with type 2 diabetes. It was found that a total of 4.1% of patients were potentially overtreated, whereas 7.8% were potentially undertreated, and 11% did not receive an HbA1c measurement (Tran et al., 2021).

Since POCT methods are already widespread in Europe, proficiency testing helps to enhance the quality of the used devices. In particular, for POCT methods measuring HbA1c, there is still room for improvement. A study by Lenters-Westra et al. (2014) showed that not all HbA1c POCT devices met the generally accepted performance criteria. In order to assess the quality class of new POCT devices, efforts should be undertaken for an IFCC standardized comparison method, the adaptation of performance to clinical conditions, and an obligation to register and participate in EQA for proof of quality and quality assurance (Lenters-Westra and English, 2019).

Another study on glucose POCT showed that participants being rated as “failed” in an EQA distribution changed devices more frequently and were, therefore, able to subsequently achieve better analytical results (Bietenbeck et al., 2018).

### Conclusion and outlook

Even if the retrospective data analysis only indicates, there appears to be a correlation between lower diabetes mortality, better glycemic control, and increased diabetes prevalence in Germany and consistently high-quality laboratory analytics. This might help to attenuate the high burden of diabetes in terms of its adverse health effects on those affected, but also in terms of its economic impacts on the global healthcare systems. Our assessment of EQA data over time can also be a valuable tool for monitoring the analytical quality of clinical chemistry parameters. It might help to raise the awareness of laboratory professionals for quality concerns.

Good laboratory diagnostics reduce the morbidity and mortality of diseased patients. Yet, diabetes monitoring technology is still on the rise. It is becoming increasingly indicated that patients with insulin-depending diabetes use CGM systems nowadays. The American Association of Clinical Endocrinology Clinical Practice Guideline from 2021 recommends the use of advanced technology in the management of people with diabetes to effectively achieve the glycemic targets, thereby improving quality and convenience of life, reducing the burden of care, and offering a personalized approach to self-testing (Grunberger et al., 2021). However, quality assurance measures comparable to the EQA protocols described in this study still need to be established internationally.

Laboratory diagnostics can also help detect patients with slowly progressive late-onset autoimmune diabetes in adults (LADA). Anti-islet autoantibodies to insulin (IAA), glutamic acid decarboxylase (GADA), tyrosine phosphatase-like protein IA-2 (IA-2A), and zinc transporter 8 (ZnT8A) are currently employed in the improved diagnostic process (Kawasaki, 2023). Here, too, EQA programs have already been established in Germany by both accredited EQA organizations.

## Data Availability

The data analyzed in this study is subject to the following licenses/restrictions: The data of the investigated EQA schemes are provided by INSTAND as excel sheets and are stored in the institute. Requests to access these datasets should be directed to p.luppa@tum.de.
