# Supplementary material for: Quality assessment of glucose measurement with regard to epidemiology and clinical management of diabetes mellitus in Germany
Source: Front Mol Biosci. 2024 Mar 20;11:1371426. doi: 10.3389/fmolb.2024.1371426 (PMC10987728; doi:10.3389/fmolb.2024.1371426)

Supplementary Material for the manuscript "Quality Assessment of Glucose Measurement with Regard to Epidemiology and Clinical Management of Diabetes Mellitus in Germany"

Peter B. Luppa, Michael Zeller, Marija Pieper, Patricia Kaiser, Nathalie Weiss, Laura Vierbaum, and Guido Freckmann

Figure 1S shows the development of the number of participants for the both German EQA organizations RfB and INSTAND over the observation period.

Figures 2S – 6S depict the box-and-whisker plots of the annual result spreads for the INSTAND EQA schemes #100, #800 and #145, given as z-values.

Description of the box-and-whisker plot: The middle line represents the median, whereas the x in the box represents the mean of the z-values. The box includes the lower and the upper quartiles (25 – 75%). The whiskers show the minimum and maximum values (± 1.5 x IQR).

Fig. 1S: Number of participants in the EQA schemes for glucose (POCT) and HbA1c from 2010 to 2022. **a+b:** RfB; **c+d:** INSTAND. Significant increases can be seen for **a**, **b** and **c**.

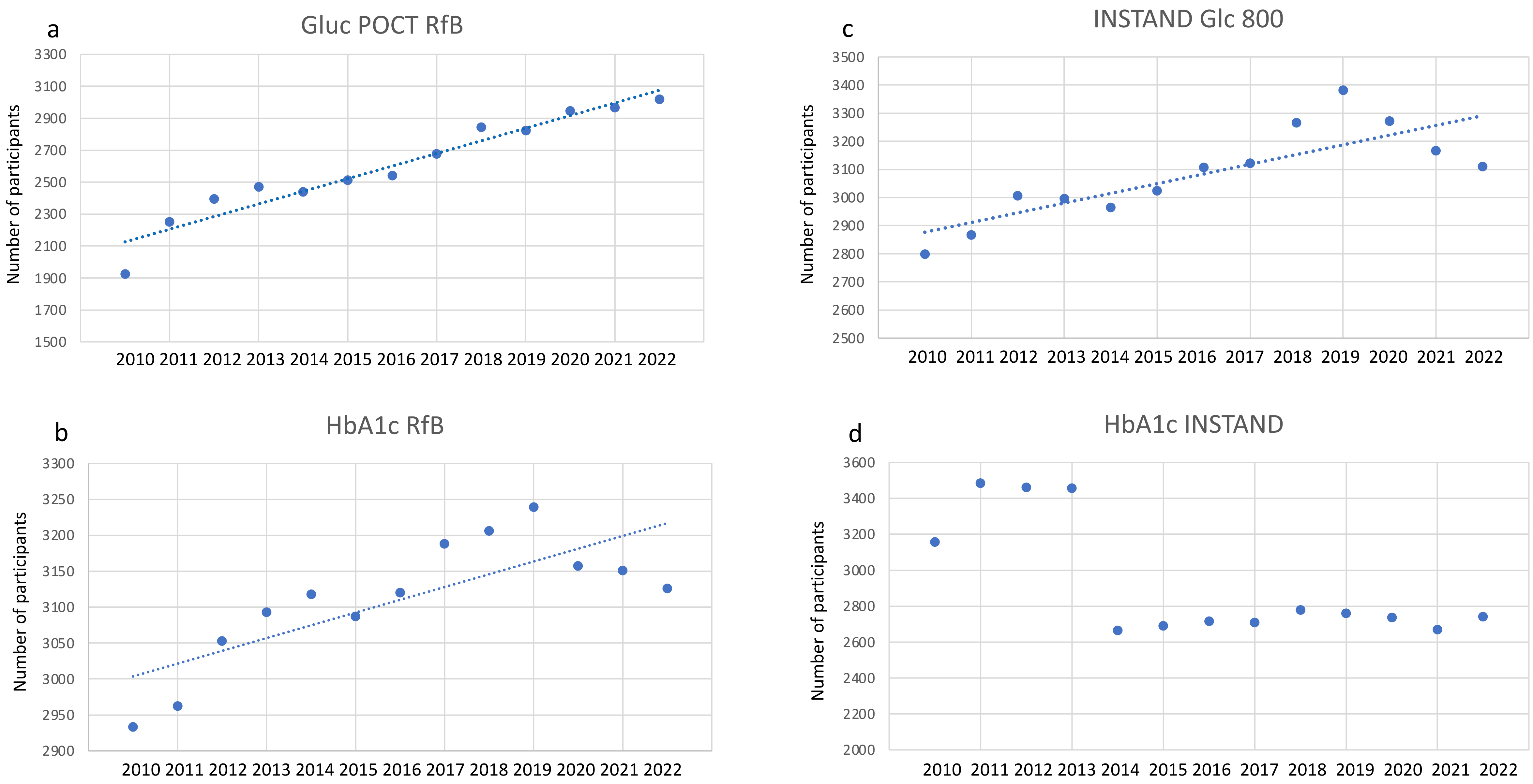

Fig. 2S: Box-and-whisker-plots of the result spreads for the INSTAND EQA scheme glucose #100, given as z-values; samples A (yellow) and B (blue)

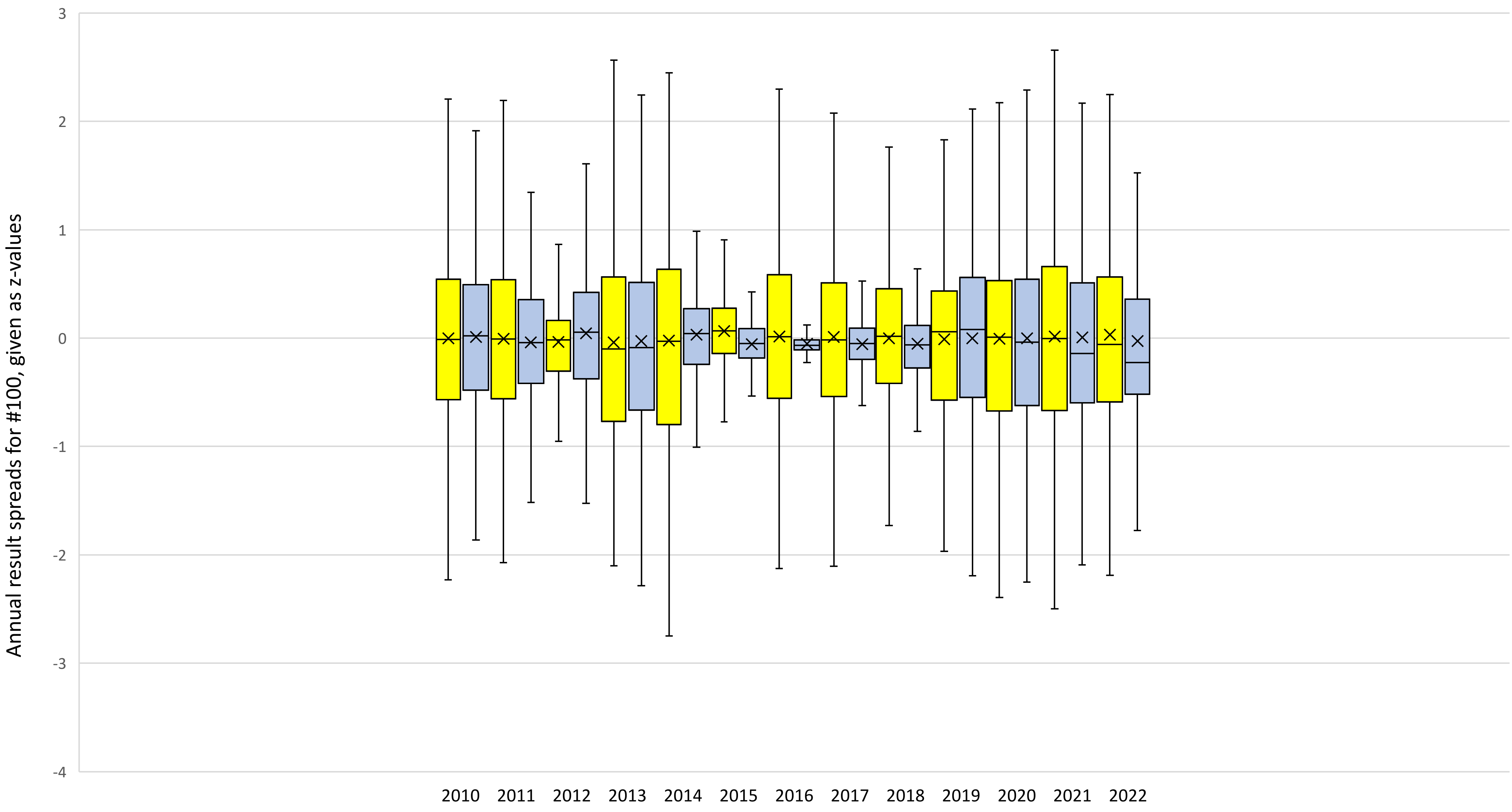

Fig. 3S: Box-and-whisker-plots of the result spreads for the INSTAND EQA scheme glucose #800, given as z-values; samples A (yellow) and B (blue)

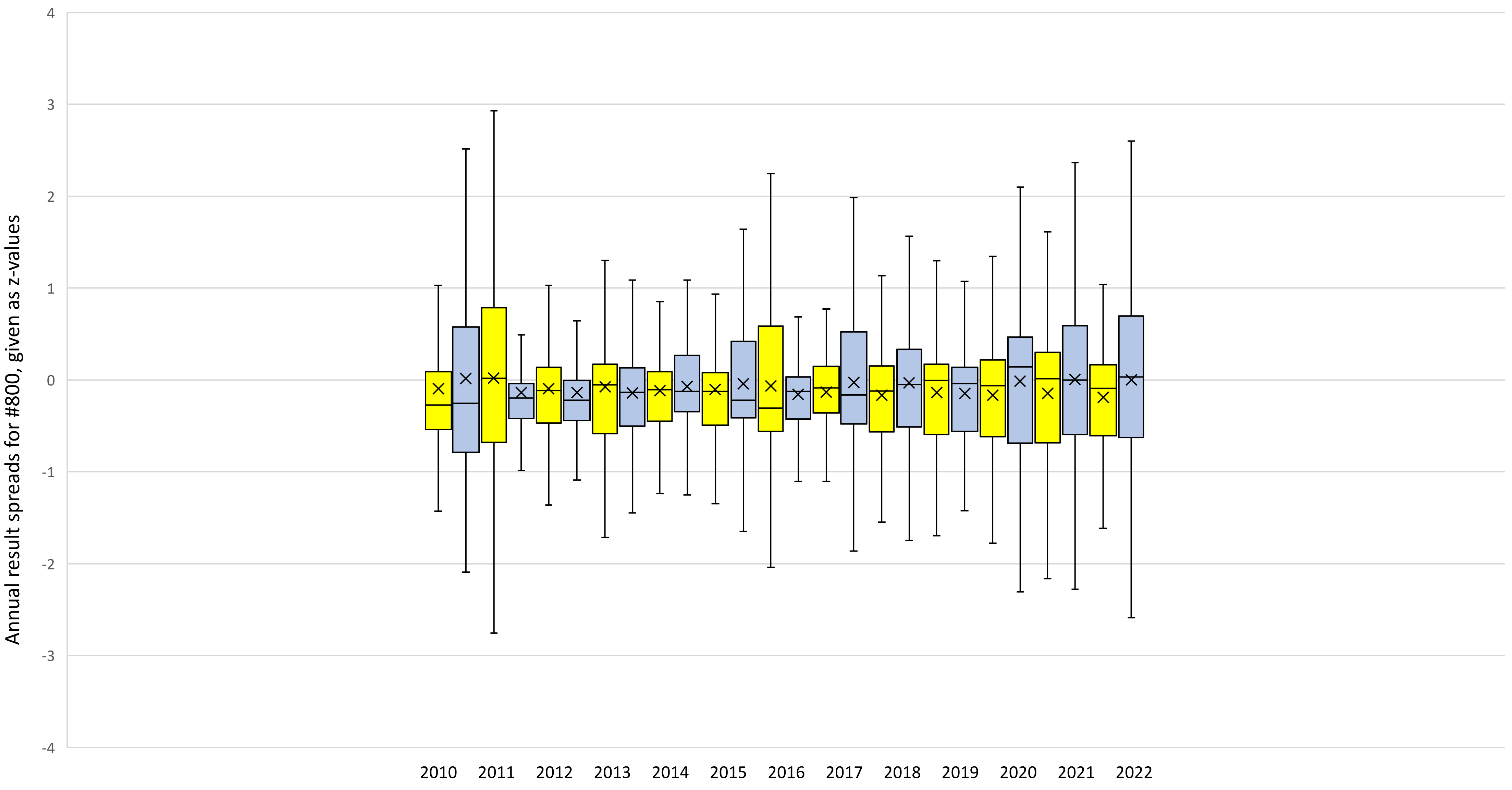

Fig. 4S: Box-and-whisker-plots of the result spreads for the INSTAND EQA scheme HbA1c #145, affinity chromatography, given as z-values; samples A (yellow) and B (blue)

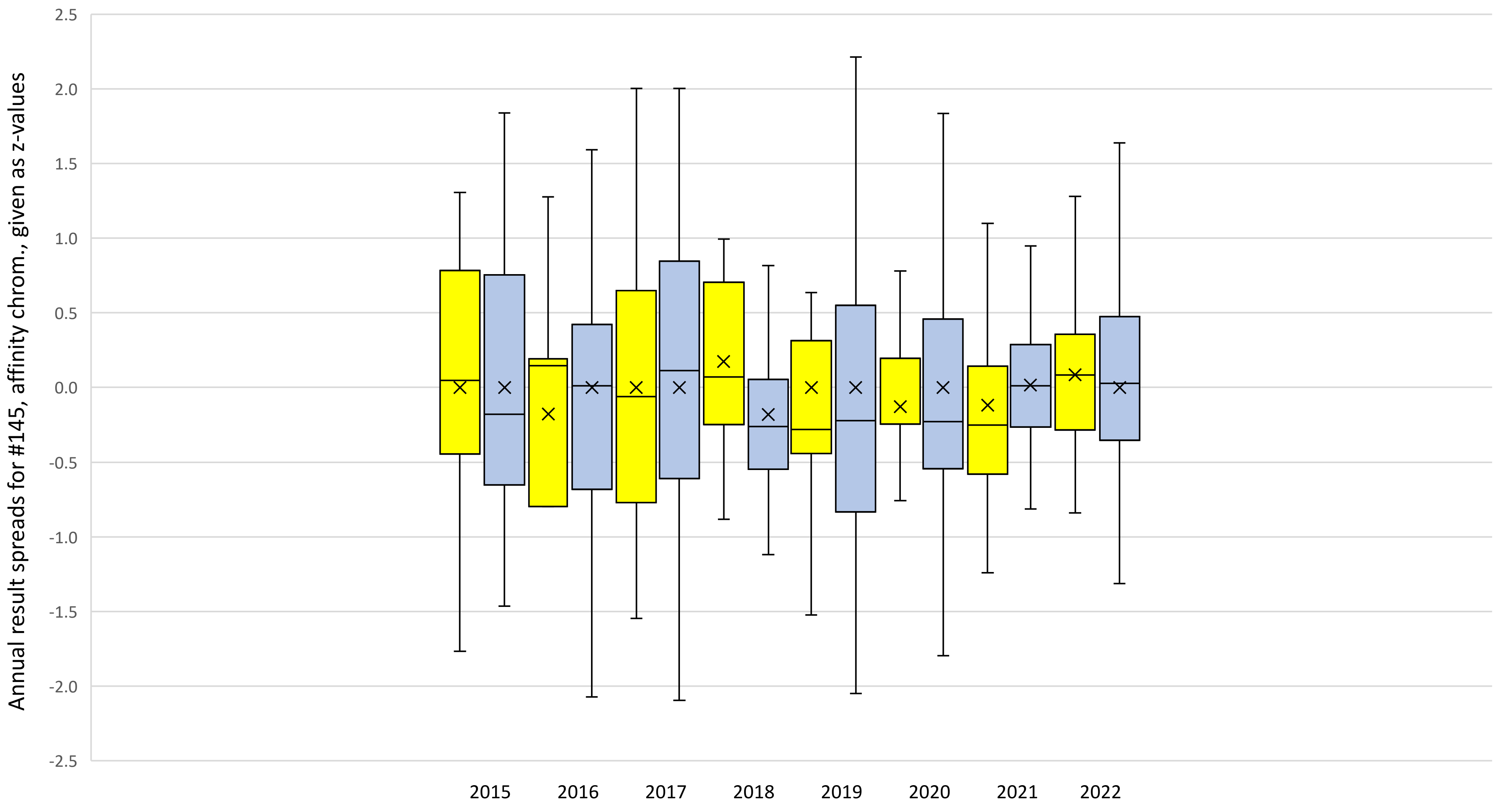

Fig. 5S: Box-and-whisker-plots of the result spreads for the INSTAND EQA scheme HbA1c #145, ion-exchange HPLC, given as z-values; samples A (yellow) and B (blue)

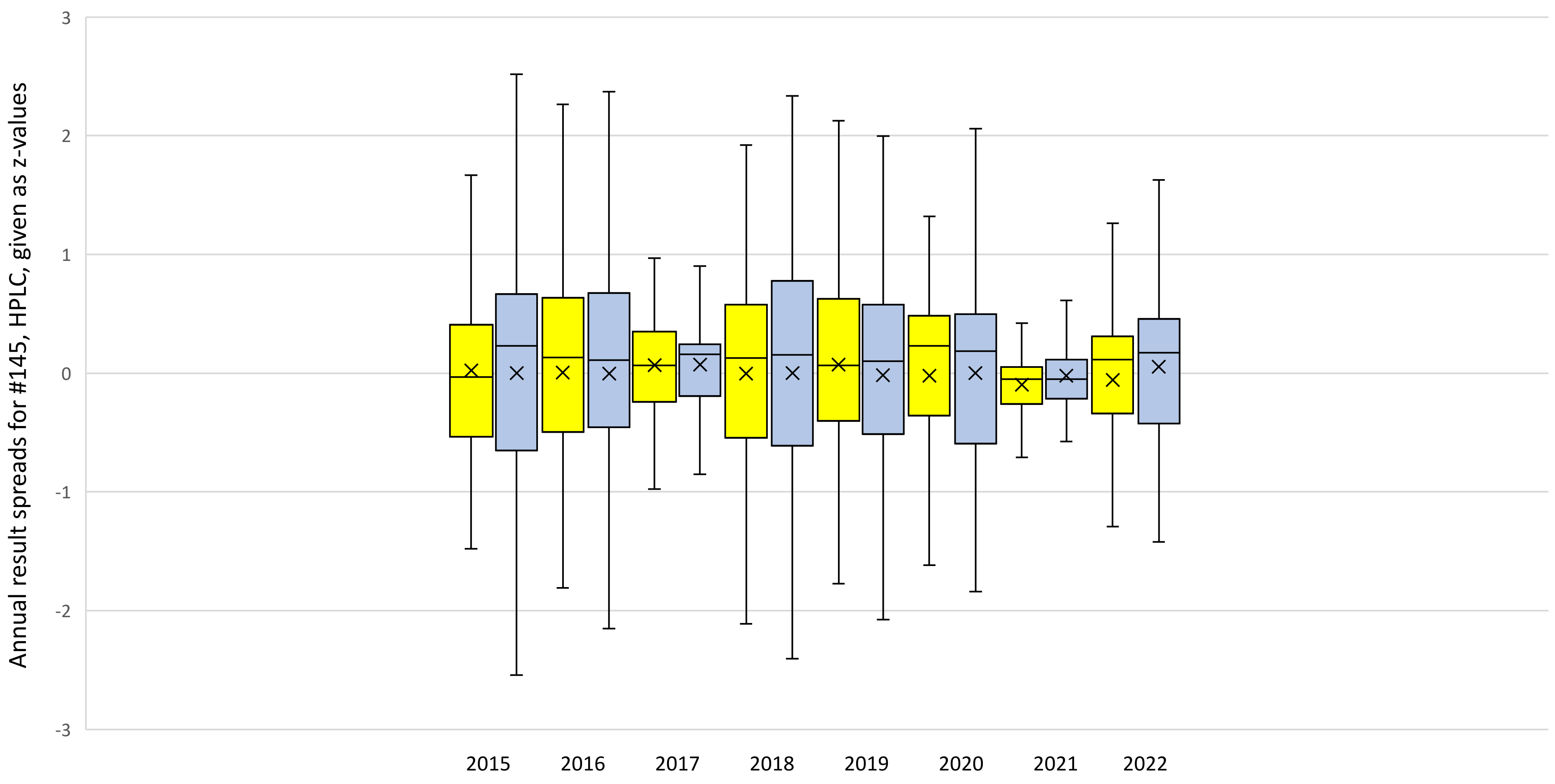

Fig. 6S: Box-and-whisker-plots of the result spreads for the INSTAND EQA scheme HbA1c #145, immunological methods, given as z-values; samples A (yellow) and B (blue)

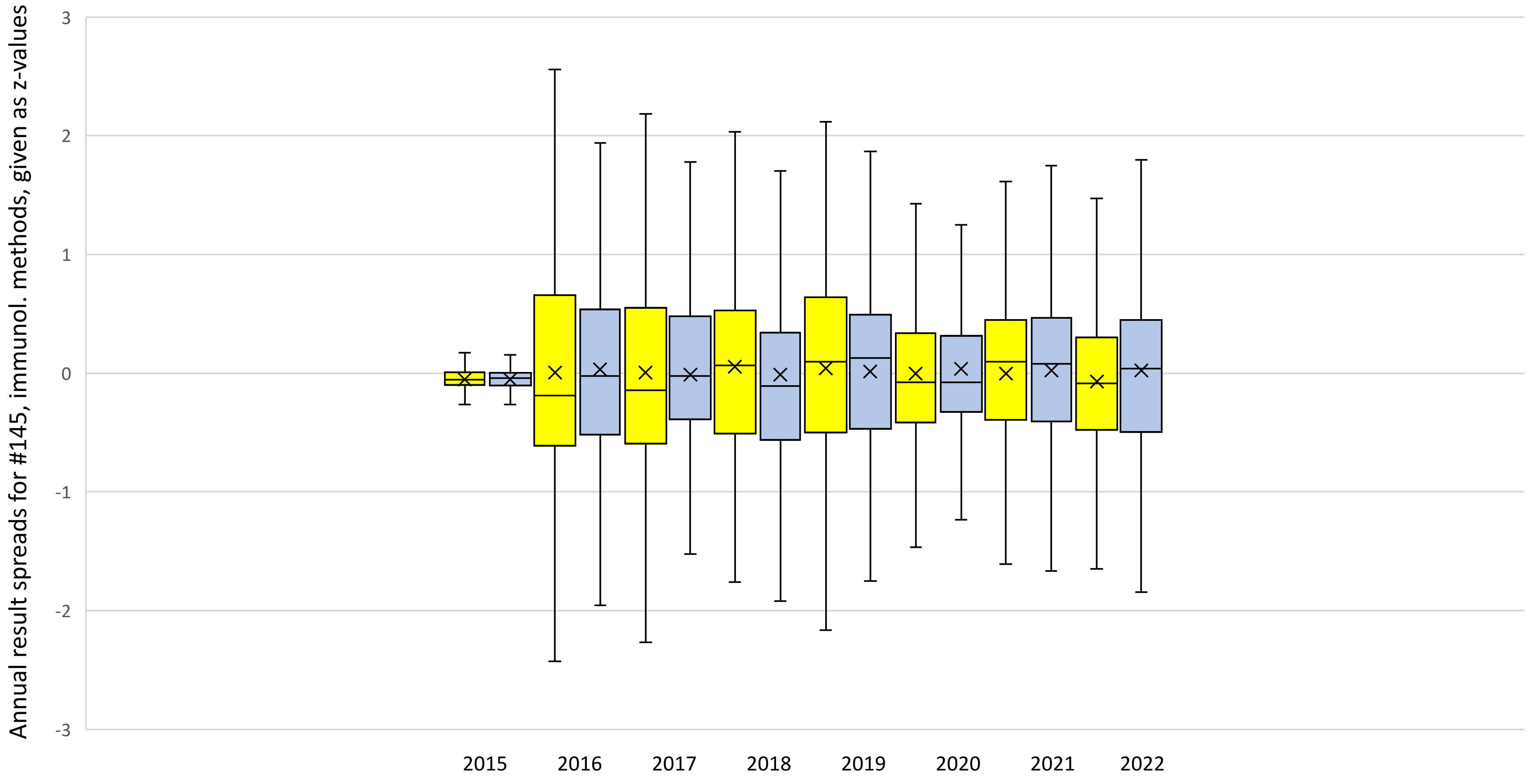

Supplement: Supplementary file 1 [file DataSheet1.PDF]
